# Obesity and Dyslipidemia in South Asians

**DOI:** 10.3390/nu5072708

**Published:** 2013-07-16

**Authors:** Anoop Misra, Usha Shrivastava

**Affiliations:** 1 Fortis-C-DOC Centre of Excellence for Diabetes, Metabolic Diseases and Endocrinology, B-16, Chirag Enclave, New Delhi 110048, India; 2 Diabetes Foundation (India), New Delhi 110016, India; 3 National Diabetes, Obesity and Cholesterol Foundation (N-DOC), New Delhi 110016, India; E-Mail: usha.shrivast@gmail.com

**Keywords:** obesity, abdominal obesity, dyslipidemia, triglycerides, Asian Indians, high density lipoprotein cholesterol (HDL), South Asians, nutrition, physical activity, type 2 diabetes

## Abstract

Obesity and dyslipidemia are emerging as major public health challenges in South Asian countries. The prevalence of obesity is more in urban areas than rural, and women are more affected than men. Further, obesity in childhood and adolescents is rising rapidly. Obesity in South Asians has characteristic features: high prevalence of abdominal obesity, with more intra-abdominal and truncal subcutaneous adiposity than white Caucasians. In addition, there is greater accumulation of fat at “ectopic” sites, namely the liver and skeletal muscles. All these features lead to higher magnitude of insulin resistance, and its concomitant metabolic disorders (the metabolic syndrome) including atherogenic dyslipidemia. Because of the occurrence of type 2 diabetes, dyslipidemia and other cardiovascular morbidities at a lower range of body mass index (BMI) and waist circumference (WC), it is proposed that cut-offs for both measures of obesity should be lower (BMI 23–24.9 kg/m^2^ for overweight and ≥25 kg/m^2^ for obesity, WC ≥80 cm for women and ≥90 cm for men for abdominal obesity) for South Asians, and a consensus guideline for these revised measures has been developed for Asian Indians. Increasing obesity and dyslipidemia in South Asians is primarily driven by nutrition, lifestyle and demographic transitions, increasingly faulty diets and physical inactivity, in the background of genetic predisposition. Dietary guidelines for prevention of obesity and diabetes, and physical activity guidelines for Asian Indians are now available. Intervention programs with emphasis on improving knowledge, attitude and practices regarding healthy nutrition, physical activity and stress management need to be implemented. Evidence for successful intervention program for prevention of childhood obesity and for prevention of diabetes is available for Asian Indians, and could be applied to all South Asian countries with similar cultural and lifestyle profiles. Finally, more research on pathophysiology, guidelines for cut-offs, and culturally-specific lifestyle management of obesity, dyslipidemia and the metabolic syndrome are needed for South Asians.

## 1. Introduction

Non-communicable diseases (NCDs) are emerging as a major health challenge in South Asians, which encompass residents of India, Pakistan, Bangladesh, Sri Lanka, Nepal, Bhutan and Maldives, constituting 24% of the world’s population [1]. According to the World Health Organization, NCDs including type 2 diabetes mellitus (T2DM), cardiovascular diseases (CVDs), chronic obstructive airways disease (COPD), cancer, injuries and mental disorders are the cause of 52% mortality, and are going to account for 72% of total mortality by 2030 in South Asia [2,3,4,5].

Globally, prevalence of obesity has doubled in the last two decades. In 2008, more than 1.6 billion adults over 20 years were overweight, of these, over 200 million men and nearly 300 million women were obese [6,7,8]. About 44% of the diabetes burden and 23% of the CVD burden is attributable to overweight and obesity; and mortality due to obesity occurs in 2.8 million adults each year [7,9,10,11,12,13]. Further, more than 43 million children under the age of five were overweight in 2010 [6]. It has been observed that 65% of the world’s population lives in countries where overweight and obesity are responsible for higher mortality than is underweight.

Obesity is associated with several co-morbid conditions: dyslipidemia, hypertension, hyperglycemia, non-alcoholic fatty liver disease (NAFLD) and a conglomeration of conditions known as the metabolic syndrome. Almost one third of the population of developed countries is detected to be having dyslipidemia [14,15]; however, prevalence varies depending on ethnic group studied. There is a wide variation in the prevalence of dyslipidemia in India depending on habitat, socioeconomic stratum and lifestyle practices [16].

## 2. Definitions

“South Asians” denote residents of India, Pakistan, Bangladesh, Sri Lanka, Nepal, Bhutan and Maldives constituting 24% of the world’s population. “Asian Indians” is a term used by authors to denote people with Indian origin. The U.S. Census Bureau uses the term Asian Indian to avoid confusion with the indigenous peoples of the Americas commonly referred to as American Indians. A “slum”, for the purpose of Census, has been defined as residential areas where dwellings are unfit for human habitation by reasons of dilapidation, overcrowding, faulty arrangements and design of such buildings, narrowness or faulty arrangement of street, lack of ventilation, light, or sanitation facilities or any combination of these factors which are detrimental to the safety and health [17].

## 3. Search Strategy

The medical search engines, Pub med (National Library of Medicine, Bethesda, MD, USA) and Google Scholar; and Governmental websites of South Asians were used for literature search using the key words, “Obesity, abdominal obesity, overweight, dyslipidemia, type 2 diabetes mellitus, insulin resistance, coronary heart disease, cholesterol, triglycerides, low density lipoprotein cholesterol (LDL), high density lipoprotein cholesterol (HDL), adipose tissue, non-alcoholic fatty liver disease, intra myocellular lipids, adipocyte, South Asians, and Asian Indians” from 1966 to December 2012. A total of 1024 references were extracted and studied. A total of 147 relevant references are being quoted. The references include reports from international and national organizations [18], chapters from books [3], review articles [19], cross-sectional studies [20], prospective studies [9] and intervention trials [7].

## 4. Guidelines for Diagnosis of Obesity

Various studies have shown that South Asians are at risk of developing obesity related co-morbidities at lower levels of body mass index (BMI) and waist circumference (WC), and that they have higher body fat at a given value of BMI than white Caucasians [21,22,23,24]. Based on these reports, it has been debated whether BMI cut-offs for diagnosis of overweight and obesity should be lower for Asian populations as compared to the available international guidelines [9,23,24,25,26,27]. In 2004, a WHO expert Consultative Committee opined that Asian populations have different associations between BMI, percentage of body fat and health risks than do European populations and suggested BMI cut-offs as ≥23–24.9 kg/m^2^ and ≥25 kg/m^2^ for overweight and obesity, respectively [26]. Subsequently, this issue has been intensively debated [24,25,27]. In an article, the WHO group discussed that no firm action should be taken internationally, and left the decision for guidelines for BMI to the governments of respective Asian countries at that time [18].

Subsequently, a Consensus Group from India formulated revised guidelines for BMI for Asian Indians (Table 1) [28]. Similar to BMI related data, cardiovascular morbidities occur at lower value of WC in Asian Indians [27], and most of the researchers have felt a need to revise international guidelines for WC for South Asians. It is important to note that International Diabetes Federation (IDF) and National Cholesterol Education Program, Adults Treatment Panel III (NCEP, ATP III) in their recent definitions of the metabolic syndrome have taken the ethnic-specific cut-off points for WC into consideration [23,24,25,29,30,31].

**Table 1:** Cut-offs of obesity and abdominal obesity for Asian Indians *vs.* international criteria.

Variable	Consensus guidelines for Asian Indians ^a^	Prevalent International Criteria
Generalized obesity	Normal: 18.0–22.9	Normal: 18.5–24.9 ^b^
(BMI cut-offs in kg/m^2^)	Overweight: 23.0–24.9 Obesity: *>*25	Overweight: 25.0–29.9 ^b^ Obesity: *>*30 ^b^
Abdominal obesity (Waist circumference cut-offs in cm)	Men: *>*90 ^c^ Women: *>*80 ^c^	Men: *>*102 ^d^ Women: *>*88 ^d^

Notes: ^a^ From Consensus guidelines for Asian Indians [28]; ^b^ According to World Health Organization guidelines [32]; ^c^ Both as per Consensus Guidelines for Asian Indians [28] and International Diabetes Federation [33]; ^d^ According to Modified National Cholesterol Education Program, Adult Treatment Panel III guidelines [34]; Adapted from [27].

## 5. Prevalence

### 5.1. Generalized Obesity

The prevalence of obesity in South Asians varies according to the age, gender, place of residence, socio-economic status, and criteria used for the measurement of obesity. In general, increasing prevalence of obesity has been seen in all studies carried out in India and other South Asian countries as shown in Table 2.

**Table 2:** Prevalence of obesity in South Asians.

Studies	Location in India/urban/rural	Age (year)	Sample population (men)	Sample population (women)	Criteria (BMI in kg/m^2^ and WC in cm)	Prevalence (%) in men	Prevalence (%) in women
Dhurandhar *et al*., 1992 [35]	West India (Urban)	>15	791	791	BMI: *>*30	4.8	7.8
Gupta *et al.*, 2003 [36]	North India (Urban)	≥20	532	559	WC: >102 (M), ≥88 (F)	21.8	44.0
Misra *et al.*, 2001 [10]	North India(Urban) **	>18	170	362	BMI: *>*25	13.3	15.6
Gupta *et al.*, 2004 [37]	North India (Urban)	>20	960	840	WC: ≥102 (M); ≥88 (F)	25.6	44.0
Prabhakaran *et al.*, 2005 [38]	North Indian(industrial population)	20–59	2935 *		BMI: *>*25 WC: ≥90 (M); ≥80 (F)	35.0 *43.0 *	
Misra *et al.*, 2005 [39]	North India(Urban)	38.9	640 *		WC: ≥90 (M); ≥80 (F)	10.1	25.9
Gupta *et al.*, 2007 [40]	North India(Urban) ***	Mean: 43.2 (M) 44.7 (F)	226	232	BMI: ≥30, WC: ≥102 (M); ≥88 (F)	20.8	34.5
Deepa *et al*., 2007 [41]	South India(Urban)	>20	2350 *		BMI: ≥25 WC: ≥90 (M); ≥80 (F)	43.256.2	47.435.1
Wijewardene *et al.*, 2008 [42]	Sri Lanka	30–65	2692	3355	BMI: ≥25	20.3	36.5
Zaman *et al.*, 2001 [43]	Bangladesh (Rural)	>18	238	272	WC: ≥94 (M); ≥80 (F)	2.9	16.8
Nanan [44]	Pakistan	25–64	National Survey		BMI: >30	13	23
Vaidya *et al.*, 2008 [45,46]	Kathmandu, Nepal (urban)	21–57	341		BMI: >25	33	
Chow *et al.*, 2008 [47]	South India(rural)	20–90	4535 *		BMI: >25 WC: ≥90 (M); ≥80 (F)	32.4	41.4
Bhardwaj *et al.*, 2011 [48]	North India (urban)	>18	217	242	BMI: >25, WC: ≥90 (M); ≥80 (F)	50.2	50
Gupta *et al.*, 2011 [49]	West India (Urban)	35–70	4621		WC: ≥102 (M); ≥88 (F)	14.4	

Notes: * Overall including male and female; ** Data from urban slum population of New Delhi, north India; *** Data from Punjabi Bhatia community in north India; M, Male; F, Female; BMI, Body mass index; WC, Waist circumference; Adapted from [9].

There are some countries in South Asia with low mean BMI values, e.g., Bangladesh (estimated mean BMI in 2008 was less than 21 kg/m^2^). However, the recent trends indicate increase in the prevalence of overweight and obesity since 1990s even in Bangladesh and Nepal [50] (Table 2).

### 5.2. Urban

The prevalence rates of obesity are higher in urban areas as compared to the rural areas, since these are most affected by rapid changes in nutritional pattern and sedentary life style [9,27,51]. In 2003, the prevalence of obesity was 13.5% (10% in men and 15.1% in women) in New Delhi (north India) [10]. In 2004, overweight (BMI ≥ 25 kg/m^2^) was present in 54.0% men and 69.4% women, while obesity (BMI ≥ 30 kg/m^2^) was present in 20.8% men and 32.3% women in Jaipur city in Western India [19]. In a recent study by our group on urban adult population in New Delhi (north India), the prevalence of obesity was observed to be 50.1% (50.2% in males and 50.0% in females) [48].

### 5.3. Rural

In 1997, prevalence of obesity was reported as 8% in rural north India [52]. However, in 2008, in rural areas in Andhra Pradesh in south India, the prevalence figures for overweight were 32.4% in men and 41.4% in women using Asian cut offs [47]. In a study in rural Tamilnadu (south India) in 2012 using Asian cut offs for obesity, the prevalence was reported to be as high as 32.8% in males and 38.2% in females [53].

### 5.4. Abdominal Obesity

Various studies have shown high prevalence of abdominal obesity in South Asians. In this ethnic group, abdominal obesity has been recognized as an important risk factor for T2DM, the metabolic syndrome and CVD [9,54,55,56]. In southern part of India (Tamilnadu), using Asian cut offs, abdominal obesity was present in 17.6% males and 23.7% females [53]. In a recent study on urban population of Delhi, abdominal obesity was observed in 68.9% subjects (62.2% males and 74.8% females) [48]. A high prevalence of abdominal obesity was shown even in underprivileged population of urban slums as well [10].

### 5.5. Obesity in Women

The available data suggest that South Asian women are comparatively more obese than men. The rise in the prevalence of overweight and obesity in women in India is reflected in the report of the National Family Health Survey (NHFS; NHFS-2 (1998–1999; conducted on 90,000 women in the age group of 15–49 years in 26 states), and NHFS-3 (2005–2006, conducted on 124,385 women in 29 states) [57,58]. The NHFS-3 data (WHO cut offs for obesity used) showed that 24% of urban women were obese as compared to 9.4% in NFHS-2. In rural areas, the prevalence has risen from 2.6% in NFHS-2 to 7% in NFHS-3 [57,58,59]. These women belonged to different socio-economic strata, and had varied educational and occupational backgrounds and differing caste, community and religion. According to our previous study on post-menopausal women residing in urban slums in New Delhi, overweight and abdominal obesity were present in 23.7% and 28% of women, respectively [10]. In a recent multi-site study conducted on 4608 women over 35 years of age in India, using WHO cut offs, 33.2% rural women were overweight as compared to 46.6% of the urban women as shown in Table 2 [49].

### 5.6. Obesity in Children/Adolescents

There is a wide variation in the prevalence data for childhood obesity globally. In 2010, 43 million children (35 million in developing countries) were estimated to be overweight and 92 million were at risk of being overweight [6]. The worldwide prevalence of childhood overweight and obesity increased from 4.2% (95% CI: 3.2%, 5.2%) in 1990 to 6.7% (95% CI: 5.6%, 7.7%) in 2010. Data from many developing countries show an increase in prevalence of obesity in children and adolescents [60,61,62,63,64,65,66].

The prevalence of overweight/obesity in urban post-pubertal children in Delhi showed an increase from 16% in 2002 to about 24% in 2006 [63]. A high prevalence was seen in the private schools (catering to children from upper socio-economic stratum) 29% *vs.* 11% in government schools (catering to lower socio-economic stratum) [60,61,62,63,64,65,66]. In a recent study by our group conducted on nearly 38,000 children across five Indian cities: New Delhi, Jaipur, Agra (north), Allahabad (central) and Mumbai (west), prevalence of overweight was 14.4% and obesity 2.8% according to International Obesity Task Force cutoffs [66]. Further, abdominal obesity was higher in girls than in boys (*p* < 0.001), being the highest in 8-year-old females (18.6%) [63,64,65,66]. Secular trends were observed among urban north Indian adolescents over a period of 5 years (2003–2008). A significant increase in WC (7.2 cm, *p* < 0.0001), W-HR (0.8, *p* < 0.0001), triceps skinfold thickness (6.5 mm; *p* < 0.0001) and FBG (3.5 mg/dL, *p* < 0.04) was noted in girls, while a significant decrease was noted for HDL-c (−4.6 mg/dL, *p* < 0.0002) in boys [63].

Overall, there has been a general trend of increase of obesity in South Asia, including women and children [9,10,11,60,61,62,63,64,65,66]. Clearly, there is an urgent need for further epidemiological research using uniform criteria and standardized methodology for the diagnosis of obesity. Further, the studies should include populations from all regions and all sections of populations for generation of valid prevalence data from South Asian countries.

## 6. Phenotype of Obesity in South Asians

Various studies have shown that obesity phenotype differs according to ethnicity. In South Asians, several features of body composition are different from those seen in white Caucasians [9,20,27,67,68,69,70,71,72].

### 6.1. Body Fat

Several studies have shown that at similar level of BMI, body fat level is higher in Asians, particularly South Asians, as compared to white Caucasians [27,73,74,75,76]. This feature has been documented in other Asian ethnicities as well; Indonesians, Chinese, and Malays in Singapore [20,67,68]. In a study conducted in Singapore, at any given percentage of body fat, BMI value of Chinese, Malays and Asian Indians was 3 kg/m^2^ lower than that in white Caucasians [67,68]. This can be partly explained by ethnicity, body frame (trunk-to-leg-length ratio and lean body), muscularity and adaptation to chronic calorie deprivation [77].

### 6.2. Truncal and Abdominal Adiposity

The truncal fat includes fat over chest and abdomen both subcutaneous abdominal adipose tissue (SCAT) and intra-abdominal adipose tissue (IAAT), all of which are more in Asian Indians than in white Caucasians [9,27,75,76]. The metabolic perturbations and adverse cardiovascular risk may be associated more with fat deposition in specific location over trunk and abdomen.

Migrant Asian Indians in USA had significantly greater total abdominal fat and IAAT than white Caucasians [74,75]. Further, truncal skin fold thicknesses are more in South Asians than in white Caucasians indicating more truncal SCAT [74,77,78,79,80,81] which could probably explain the higher prevalence of insulin resistance in a BMI and body fat-matched Asian Indian men as compared to white Caucasians in USA [79,81]. We have reported significant association of truncal skinfold thickness (signifying high truncal SCAT) with fasting hyperinsulinemia in Asian Indian children and adolescents as well [27]. Importantly, and contrary to results of studies on white Caucasians, SCAT was better correlated to the metabolic syndrome than IAAT in adult urban Asian Indians in multivariate analysis [82,83,84,85,86,87,88,89,90].

## 7. Prediction Equations for Insulin Resistance and Body Fat Depots for Asian Indians

Since it is not always possible to quantify insulin resistance, body fat, or body fat depots with the use of expensive and mostly hospital based measurement methods (e.g., hydro-densitometry and magnetic resonance imaging), we have developed predictive equations which use simple clinical measures. Models have been prepared using Classification and Regression Tree (CART) and multivariate regression for insulin resistance and body fat depots:

### 7.1. For Insulin Resistance

Three simple decision models have been developed based on routine clinical and biochemical parameters using CART and multivariate logistic regression to predict insulin resistance in apparently healthy Asian Indian adolescents [82,83,84]. Since costs of investigations are prohibitive at times in this region, anthropometric measurements, routine biochemical parameters, clinical parameters and gender have been used.

a. CART I, based on anthropometric parameters including ∑4SF (sum of biceps, triceps, subscapular and suprailiac skinfolds) and suprailiac skinfold thickness alone has sensitivity 88.2%, specificity 50.1% and area under Receiver Operating Characteristic curve (aROC) 77.8%.

b. CART II, based on anthropometric and routine clinical and biochemical parameters (BMI, fasting plasma glucose levels and LDL-C) has sensitivity 94.5%, specificity 38.3% and aROC 73.6%.

c. CART III, based on all anthropometric, biochemical and clinical parameters, hip circumference (HC in cm); percentage body fat (%BF); ratio of sum of central (suprailiac and sub scapular) skinfolds to peripheral (biceps and triceps) skinfolds (C-P ratio); body fat mass (FM) in kg and LDL-C showed sensitivity 70.7%, specificity 79.2% and aROC 77.4% [82,83,84].

### 7.2. For Body Fat Depots

The simplest equation for predicting %BF derived from DEXA included age, sex, BMI, triceps skinfold and WC (*R*^2^ = 84.4%). Replacing BMI with weight and height reduced the overall variance (*R*^2^ = 86.4%).

Equations for prediction of total abdominal fat (TAF), IAAT and SCAT are listed in Table 3 [82,83,84].

**Table 3:** Predictive equations for estimation of various body fat depots in Asian Indians.

Variable predictive equation
%BF: 42.42 + 0.003 × age + 7.04 × gender + 0.42 × TR sf + 0.29 × WC + 0.22 × Wt − 0.42 × Ht
TAF: −47,657.00 + 1384.11 × gender + 1466.54 × BMI + 416.10 × WC
IAAT: −238.7 + 16.9 × age + 934.18 × gender + 578.09 × BMI − 441.06 × HC + 434.2 × WC
SCAT: −49,376.4 − 17.15 × age + 1016.5 × gender + 783.3 × BMI + 466 × HC

Notes: %BF, % Body fat; gender: M = 1, F = 2; TR sf, Triceps skinfold; WC, Waist circumference; Wt, Weight; Ht, Height; BMI, Body mass index; HC, Hip circumference; TAF, Total abdominal fat; IAAT, Intra-abdominal adipose tissue; SCAT, Subcutaneous abdominal adipose tissue; Adapted from [48].

## 8. Deposition of Fat at “Ectopic” Sites

Insulin sensitivity can be affected by fat accumulation in tissues other than where it is usually deposited (“ectopic fat”); for example, liver, muscle and heart. It appears that South Asians have tendency for deposition of fat in some of these sites [27].

### 8.1. Hepatic Fat

It is now recognized that NAFLD an important component of the metabolic syndrome [91,92]. Hepatic steatosis accompanied with portal inflammation in advanced stages is termed as non-alcoholic steato-hepatitis (NASH) and may progress to hepatic fibrosis and even cirrhosis.

It is estimated that approximately one fourth of the urban population in India has NAFLD [93,94,95]. In a case-control study, we showed that Asian Indians in north India with NAFLD have higher adiposity, fasting hyperinsulinemia, the metabolic syndrome and glucose intolerance than those without NAFLD [93]. Further, we studied hepatic gluconeogenesis pathway in non-diabetic Asian Indian males having NAFLD using *in vivo* (^31^P) phosphorous magnetic resonance spectroscopy (MRS) and correlated it with anthropometry and surrogate marker of insulin resistance. Interestingly, non-obese non-diabetic subjects with NAFLD showed more derangements of hepatic gluconeogenesis enzymes than non-obese subjects without NAFLD [94]. In a comparative study in USA, South Asians had higher hepatic triglycerides levels, which were associated with lower adiponectin levels than white Caucasians [95,96,97]. It is possible, therefore, that Asian Indians have greater triglyceride deposition in liver than white Caucasians, which may be related to higher magnitude of insulin resistance or inherent genetic tendency.

### 8.2. Skeletal Muscle Triglycerides

Intra-myocellular lipids (IMCL) are located in the mitochondria, along with enzymes involved in fatty acid esterification, hydrolysis, ion transport and cellular oxidation. Combined effect of high concentration of serum insulin and free fatty acids can cause enhanced storage of IMCL. These lipids are believed to be important in the pathogenesis of insulin resistance and can be measured non-invasively using proton MRS [94,96,97]. We previously showed that excess IMCL deposition in soleus muscle was associated with abdominal obesity, but unlike in case of white Caucasians, the correlation with fasting insulin levels was not observed [98,99,100]. In a study by our group, the soleus muscle IMCL content and high sensitivity C-reactive protein (hs-CRP) levels were significantly higher in T2DM patients compared to healthy controls. However, values of insulin, other measures of insulin resistance, and hs-CRP levels did not correlate with soleus muscle IMCL content [98,99,100].

### 8.3. Other Ectopic Sites of Fat Deposition

There are few other ectopic sites of fat deposition; over the neck (“buffalo hump”) frequently observed in Cushing’s syndrome and HIV-associated lipodystrophy, and excess fat under the chin (“double chin”) seen in familial partial lipodystrophy [101]. These ectopic fat depositions are frequently associated with insulin resistance and other features of the metabolic syndrome [101,102,103]. Extending research on these signs to obese people in whom these signs are frequently seen, we showed that mild “buffalo hump”, and “double chin” signify the heightened risk of metabolic syndrome in urban Asian Indians [104].

### 8.4. Adipocyte Size

**Figure 1:**
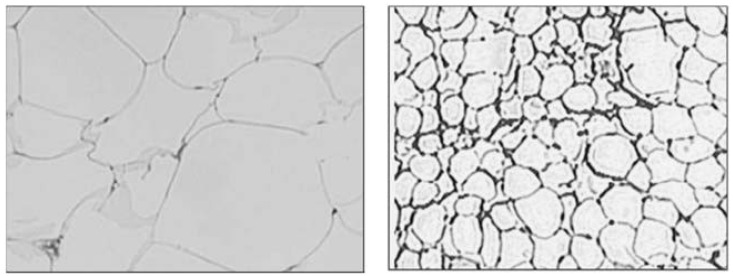
Comparative pictures of enlarged adipocytes from South Asian (left) and White (right) volunteers. Both images are obtained with SPOT digital camera using 10 magnification. Note: this figure is reproduced with permission from [74]. Copyright Chandalia *et al.*, 2007.

Large subcutaneous abdominal adipocyte size predicts insulin resistance and T2DM independent of obesity [105]. In this study, done on Pima Indians, mean subcutaneous abdominal adipocyte size was 19% and 11% higher in subjects with T2DM and impaired glucose tolerance (IGT), respectively as compared to persons with normal glucose tolerance (*p* < 0.001) [105]. In this context, it is important to note that adipocyte cell size was significantly higher in South Asians (3491 ± 1393 µm^2^ ) as compared to white Caucasians in USA (1648 ± 864 µm^2^; *p*-value = 0.0001) [74]. These findings need to be further researched for clinical implications (Figure 1).

## 9. Dyslipidemia

Dyslipidemia signifies the increased concentration of total cholesterol and LDL cholesterol, decreased concentration of HDL cholesterol and hypertriglyceridemia present alone or in combination. A combination of lipid abnormalities, hypertriglyceridemia and low HDL, are metabolically interlinked and have been termed as “atherogenic dyslipidemia” [106,107]. This pattern of dyslipidemia has shown a strong association with T2DM and CVD in several studies in developed countries [107,108].

There is a paucity of studies on dyslipidemia in South Asians. The overall prevalence of dyslipidemia in India in various studies ranges from 10% to 73% [16], depending on area of residence (rural *vs.* urban), socio-economic stratum (high *vs.* middle or low), diet and physical activity patterns and age. The prevalence of hypertriglyceridemia varied from 73% in obese and 61% in non-obese Asian Indians in urban areas and migrant Asians, the levels were relatively lower in rural areas but still higher than white Caucasians. The results from a recent cross-sectional study in urban New Delhi (north India) showed the prevalence of hypertriglyceridemia to be 42.7% [48] (Table 4). In rural areas, the prevalence figures were lower than urban areas; however, recent data show increasing prevalence [36,54]. In particular, HDL levels are lower in South Asians than in White Caucasians as shown consistently in several comparative studies (Table 5). Importantly, the prevalence of atherogenic small, dense LDL was significantly higher in Asian Indians compared with white Caucasians in USA (44% *vs.* 21%; *p* < 0.05) [109] and may contribute to increased tendency for CVD in this ethnic group. Finally, plasma concentration of adipose tissue metabolites, leptin and non-esterified fatty acids are higher and adiponectin levels are lower in insulin resistant Asian Indians as compared to more insulin sensitive Caucasians and could contribute to insulin resistance and atherogenic dyslipidemia [110,111].

**Table 4:** Prevalence of obesity and cardio-metabolic risk factors in urban population of New Delhi, India (*n* = 459).

Variable	Percentage
Obesity (BMI criteria)	50.1
Impaired Fasting Glucose	24
Diabetes	8.5
Hypercholesterolemia	26.6
Hypertriglyceridemia	42.7
LDL-C ≥ 100 mg/dL	51.6
HDL-C < 40 mg/dL (males) and <50 mg/dL (females)	37

Notes: BMI ≥ 25 kg/m^2^ defined as obesity; LDL-C = low Density lipoprotein cholesterol; HDL-C = high density lipoprotein cholesterol; Adapted from [48].

**Table 5:** Differences in the high-density lipoprotein cholesterol (HDL) levels between South Asians/Asian Indians *vs*. Whites/Europeans. Note: this table is reproduced with permission from [27]. Copyright The Endocrine Society, 2008.

Author	*N*	Parameter	South Asians/Asian Indians	Whites/Europeans
*Adults*				
Chandalia *et al*. [112]	1031 AIs and 455 Whites ^a^	Percent population ^j^ with low HDL	M, (42% ^m^; 52% ^n^); F, (56% ^m^; 72% ^n^)	M, 35%; F, 25% *
Ajjan *et al*. [113]	245 SAs and 245 UK Whites	HDL (mmol/L)	1.10	1.43 **
Williams *et al*. [114]	63 SAs and 42 Europeans ^b^	HDL (mmol/L)	1.27	1.20
Smith *et al*. [115]	82 AIs and 83 Caucasians ^c^	HDL (mmol/L)	M, 0.97; F, 1.13	M, 1.24; F, 1.51 *
Somani *et al*. [116]	141 SAs and 121 Whites	HDL (mmol/L)	1.1	1.5
Bhalodkar *et al*. [117]	119 AIs and 1752 Caucasians ^d^	HDL (mmol/L) HDL size (nm) ^k^	1.378.9	1.379.4
Forouhi *et al*. [118]	113 SAs and Caucasians ^e^	HDL (mmol/L)	M, 1.26, F, 1.51	M, 1.39; F, 1.56
Chambers *et al*. [119]	518 AIs and 507 Whites ^f^	HDL (mmol/L)	1.22	1.33 **
Enas *et al*. [120]	1131 AI men and 557 AI women compared with Caucasians from FOS ^g^	HDL (mmol/L) ^l^	M, 0.98; F, 1.24	M, 1.18 **; F, 1.45 **
McKeigue *et al*. [121]	1421 SAs and 1515 Europeans ^h^	HDL (mmol/L)	1.16	1.25
McKeigue *et al*. [122]	253 Bangladeshis and Europeans ^i^	HDL (mmol/L)Percent of TC as HDL (%)	M, 1.13; F, 1.19M, 21.3; F, 22.4	M, 1.43; F, 1.45M, 25.3; F, 25.2
*Children*				
Ehtisham *et al*. [123]	65 SAs and 64 European adolescents (14–17 years)	HDL (mmol/L)	M, 1.28; F, 1.49	M, 1.39; F, 1.67
Whincup *et al*. [124]	73 SAs and 1287 Caucasian children (10–11 years)	HDL (mmol/L)	1.38	1.43

Notes: AI, Asian Indian; BMI, body mass index; CURES, The Chennai Urban Rural Epidemiology Study; F, female; FOS, Framingham Offspring Study; HDL, high-density lipoprotein cholesterol; M, male; *N*, number of sample population; SA; South Asians; TC, total cholesterol; UK, United Kingdom; ^a^ Indigenous Asian Indians from CURES study; ^b^ Men aged 35–75 years; ^c^ Aged 20–60 years; ^d^ Women from the Framingham Offspring Study; ^e^ BMI matched, aged 40–55 years; ^f^ Aged 35–60 years; ^g^ Women from the Framingham Offspring Study; ^h^ Males; ^i^ Aged 35–69 years; ^j^ Low HDL: males < 1.036 mmol/L and females < 1.295 mmol/L; ^k^ HDL particle size in nanometers; ^l^ Men aged 30–39 years, and women aged 30–59 years; ^m^ Urban; ^n^ Rural; * *p* < 0.0001; ** *p* < 0.001.

## 10. Determinants of Obesity and Dyslipidemia in South Asians

### 10.1. Nutritional Transition

South Asians are becoming increasingly more affluent. Further, with economic liberalization, there is a widespread presence of transnational food company outlets and availability of packaged foods in 24 h supermarkets. With better purchasing power, South Asians are increasingly consuming diets high in saturated fats, cholesterol, and refined carbohydrates and low in polyunsaturated fatty acids and fiber [51]. Availability of edible vegetable oils for consumption has nearly tripled in developing countries in the last few years. Importantly, while processed non-traditional “fast-foods” contribute to faulty diets, some of the locally made “fast foods” sold by street vendors in several developing countries are equally unhealthy. These food items contain high amount of trans fatty acids (TFA) due to deep-frying using low cost and widely available partially hydrogenated vegetable oils [51,125].

### 10.2. Urbanization, Demographic Transition and Rural-to-Urban Migration

In South Asia, urbanization is increasing rapidly and is now nearly 38%, but is expected to be 50% by 2020 [126]. Urbanization exposes people to a number of challenges, imbalanced diets, physical inactivity, long working hours and other urban stress making them vulnerable to NCDs. The average life expectancy of Indian population at birth now is 67.14 years, as compared to 31 years in 1947 [127]. Similarly, life expectancy is going up in the other countries of the region as well. This has resulted in the rise of elderly population in the region, again leading to rise in NCDs.

**Figure 2:**
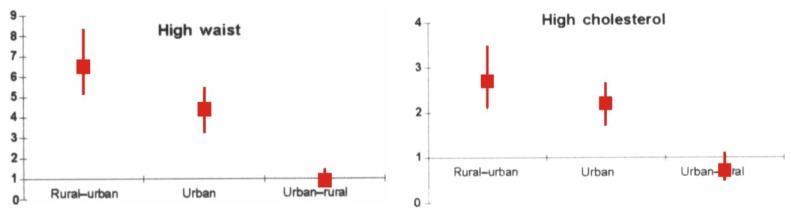
Age-adjusted ORs and 95% CIs for prevalence of cardiovascular risk factors in different groups of women (rural-urban, urban and urban-rural migrants) as compared with the rural women, high prevalence of high waist circumference ≥80 cm, and hypercholesterolemia ≥200 mg/dL among rural-urban migrants and urban women. The prevalence declines among the urban-rural migrants. Note: this figure is reproduced with permission from [49]. Copyright the BMJ Publishing Group Ltd., 2011.

Migration, whether inter-country or rural-to-urban within country, is a risk factor for T2DM. In a review, South Asian migrants showed nearly four times high prevalence rates of T2DM than those of rural sedentee populations. Similar observations were also reported in intra-country migrants and resettled indigenous populations [128]. Migration results in increasing physical inactivity, faulty nutrition and exposure to stress. We have previously shown that migrant postmenopausal women settled in urban slums have high prevalence of multiple CVD risk factors [10]. In a recent study, we have shown a gradient in NCDs between rural, rural-urban migrants and urban residents [49]. Importantly, there was a significant correlation of duration of migration with waist size and high fat content in the diets (*p* < 0.001) (Figure 2).

### 10.3. Physical Inactivity

Sedentary life style compounded with the change in the nutritional pattern in South Asians makes them more vulnerable to NCDs [27]. The changes of occupations, advent of newer technologies, and rapid pace of urban life have increasingly resulted in more sedentary work and less energy expenditure; however, this needs more research in context of South Asians. In one such study, lower levels of physical activity in Asian Indians, Pakistanis and Bangladeshis was seen to be inversely correlated with BMI, WC, systolic blood pressure, plasma glucose and insulin levels [27,129]. Determinants of physical inactivity in South Asians have not been systematically studied.

### 10.4. Socio-Economic and Cultural Factors

The prevalence of obesity, dyslipidemia, T2DM and CVD in South Asia is more in the people belonging to the upper socio-economic strata unlike in the developed nations [9,27]. However, with new found wealth and a number of dietary choices and “western foods” available at relatively low prices, people belonging to middle and low socio-economic strata are being increasingly afflicted with NCDs [27,62].

Socio-cultural and psychological factors and prevalent misconceptions are important in modifying diet and lifestyle habits of women and children in South Asia. In this region, there is a prevalent misconception that an “obese child is a healthy child” and, hence should be fed in excess. Mothers often have traditional belief that feeding excess *ghee* (clarified butter) and butter to child would be beneficial to growth and impart them strength. In a cross-sectional study of 1800 children aged 9 to 18 years and their mothers, using qualitative (focus group) and quantitative (semi-structured survey) data, widely prevalent myths, and correlation between obesity and dietary habits of children and their mothers has been shown [130]. Other social factors as a cause of physical inactivity are: priority for academics at the cost of playing time in children, increasing use of television and computers, lack of playfields and open spaces, and security concerns in the outdoors, especially for women [60,61,66]. In particular, cultural and social restrictions for outdoor physical activity in women in South Asian countries may be an important reason for increasing obesity and the metabolic syndrome.

### 10.5. Genetics

**Figure 3:**
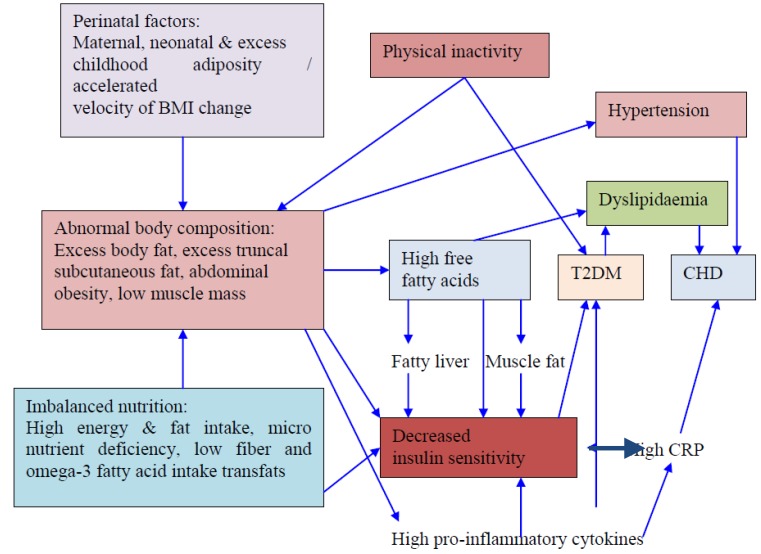
Complex interactions of genetic, perinatal, nutritional and other acquired factors in development of insulin resistance, type-2 diabetes and coronary heart disease in South Asians. T2DM, type 2 diabetes mellitus; CRP, C-reactive protein; CHD coronary heart disease. Adapted from [9].

A few studies show genetic association of obesity, insulin resistance and dyslipidemia in South Asians (Figure 3). In a recent study, variants of *Myostatin* gene was shown to predispose to obesity, abdominal obesity and low lean body mass in Asian Indians in north India [131]. In another important study, *LMNA* 1908T/T and C/T genotypes emerged as independent genetic risk factors for generalized obesity in non-diabetic Asian Indians in north India [132]. Association of *AMD1* variant with obesity has been shown in Asian Indian children [133]. Further, there is recent evidence for genetic associations of NAFLD with *SREBP-2* 1784 G>C genotype [134] and peroxisome proliferator activated receptor-γ (Pro12Ala and C161T) polymorphisms [135] in Asian Indians. A recent study has shown *DOK5* as a susceptibility gene for obesity and T2DM in Asian Indians in north India [136]. Finally, genetic susceptibility of Asian Indians to development of dyslipidemia has been shown in some studies [131,132,133,136,137,138,139].

## 11. Interventions

Low awareness regarding modifiable risk factors (diet and physical activity) in South Asians should be addressed in a comprehensive and culturally specific manner [140,141]. In this context, it is important to note that Consensus Dietary Guidelines have been developed for Asian Indians for prevention of obesity and T2DM and include reduction in the intake of carbohydrates, preferential intake of complex carbohydrates and low glycemic index foods, higher intake of fiber, lower intake of saturated fats, optimal ratio of essential fatty acids, reduction in trans fatty acids, low intake of salt and restricted intake of sugar [140]. Other document, which is important to formulate interventions, includes the Consensus Physical Activity Guidelines for Indians, where more intensive physical activity, a total of 60 min of physical activity every day, is recommended for Asian Indians. This should include at least 30 min of moderate-intensity aerobic activity, 15 min of work-related activity, and 15 min of muscle-strengthening exercises [141].

There is a paucity of well-designed intervention studies for prevention of NCDs in South Asians. The available data have been reviewed below.

### 11.1. Adults

An intervention study for prevention of T2DM, conducted in Tamilnadu (south India] on 531 males and females aged 35–55 years with impaired glucose tolerance, was designed to test lifestyle intervention compared to metformin. This study showed 28.5% reduction of incident cases of diabetes with lifestyle intervention, 26.4% with metformin and 28.2% with a combination of both as compared to the control group. Besides reiterating that lifestyle interventions are still the best for prevention of diabetes in Asian Indians, this intervention trial has demonstrated that both lifestyle and metformin interventions are cost effective strategies for prevention of diabetes in resource-constrained South Asian countries [142,143].

### 11.2. School Children

An intervention program entitled, “*M*edical education for children/*A*dolescents for *R*ealistic prevention of obesity and diabetes and for healthy a*G*eing (MARG; Hindi for “path”) conducted in three large cities of North India (New Delhi, Agra and Jaipur) resulted in novel research data in schoolchildren residing in India. A sub study, in which the knowledge and practices (KAPs) of children regarding health, nutrition and NCDs on 2500 children were assessed, these parameters were shown to be significantly improved in the post-intervention phase, particularly in 8–11 years old as compared to their elder counterparts (12–18 years). Similarly, significantly higher improvement of KAPs was recorded in children studying in government schools (catering to low socio-economic stratum) as compared to private schools (catering to high and middle socio-economic strata) [144]. In another sub-study, 15–17 years old children were researched in two randomly allocated schools (intensive education and counseling in one school *vs.* usual education and counseling in the second school). After 6 months of intervention, we observed better lifestyle practices (less TV viewing and eating more fruits), a significant decrease in waist hip ratio, better insulin sensitivity, and significantly lower hs-CRP values in the intervention group *vs.* control group [144,145,146,147]. These data provide locally and culturally specific modules for successful intervention in South Asians at an early age.

## 12. Conclusions

South Asians are facing growing “epidemics” of obesity and dyslipidemia. Several factors including rapid urbanization, demographic changes, rural-to-urban migration, faulty diets, sedentary lifestyle, socio-cultural factors alongwith genetic predisposition have emerged as major contributory factors. Obesity in south Asians showed certain distinct features including preponderance of abdominal obesity, more intra-abdominal and truncal sub-cutaneous adiposity, fat deposition in liver (fatty liver) and skeletal muscles. Different and lower cut offs for BMI and waist circumference, and specific guidelines for diet and physical activity have been advocated for Asian Indians. Evidence is available for effective intervention programs with emphasis on nutrition, physical activity and life style changes in children, and also in adults for prevention of obesity, T2DM and dyslipidemia.
